# The Two-Way Switch Role of ACE2 in the Treatment of Novel Coronavirus Pneumonia and Underlying Comorbidities

**DOI:** 10.3390/molecules26010142

**Published:** 2020-12-31

**Authors:** Xiao Cong Pang, Han Xu Zhang, Zhi Zhang, Suguro Rinkiko, Yi Min Cui, Yi Zhun Zhu

**Affiliations:** 1 School of Pharmacy and State Key Laboratory for the Quality Research of Chinese Medicine, Macau University of Science and Technology, Macau 999078, China; pangxiaocong1227@163.com (X.C.P.); suguro_apple@foxmail.com (S.R.); 2 Department of Pharmacy, Peking University First Hospital, Beijing 100034, China; zhanghanxu95@163.com; 3 School of Pharmaceutical Sciences, Tsinghua University, Beijing 100084, China; zhangz2019@mail.tsinghua.edu.cn; 4 Shanghai Key Laboratory of Bioactive Small Molecules, Department of Pharmacology, School of Pharmacy, Fudan University, Shanghai 201203, China

**Keywords:** severe acute respiratory syndrome (SARS), angiotensin-converting enzyme II (ACE2), renin-angiotensin system (RAS), lung injury, hypertension

## Abstract

December 2019 saw the emergence of the coronavirus disease 2019 (COVID-19), caused by severe acute respiratory syndrome coronavirus-2 (SARS-CoV-2), which has spread across the globe. The high infectivity and ongoing mortality of SARS-CoV-2 emphasize the demand of drug discovery. Angiotensin-converting enzyme II (ACE2) is the functional receptor for SARS-CoV-2 entry into host cells. ACE2 exists as a membrane-bound protein on major viral target pulmonary epithelial cells, and its peptidase domain (PD) interacts SARS-CoV-2 spike protein with higher affinity. Therefore, targeting ACE2 is an important pharmacological intervention for a SARS-CoV-2 infection. In this review, we described the two-way switch role of ACE2 in the treatment of novel coronavirus pneumonia and underlying comorbidities, and discussed the potential effect of the ACE inhibitor and angiotensin receptor blocker on a hypertension patient with the SARS-CoV-2 infection. In addition, we analyzed the S-protein-binding site on ACE2 and suggested that blocking hot spot-31 and hot spot-353 on ACE2 could be a therapeutic strategy for preventing the spread of SARS-CoV-2. Besides, the recombinant ACE2 protein could be another potential treatment option for SARS-CoV-2 induced acute severe lung failure. This review could provide beneficial information for the development of anti-SARS-CoV-2 agents via targeting ACE2 and the clinical usage of renin-angiotensin system (RAS) drugs for novel coronavirus pneumonia treatment.

## 1. Introduction

December 2019 saw the emergence of the coronavirus disease 2019 (COVID-19), caused by severe acute respiratory syndrome coronavirus-2 (SARS-CoV-2), which emerged in Wuhan, Hubei province, in central China, and spread rapidly across the globe [1,2]. Today, the outbreak constitutes a public health emergency of international concern (PHEIC). The SARS-CoV-2 outbreak is not only in China, but also throughout the rest of the world, as the number of infections continues to rise and the epidemic spreads to more countries. SARS-CoV-2 has a high similarity to bat coronaviruses, which has been postulated to be the primary source [3]. Current evidence suggests pangolins could be recognized as possible intermediate hosts and the transmission path of SARS-CoV-2 from pangolin to humans is being looked into [4].

Typical clinical features of SARS-CoV-2 infected patients are fever, dyspnea, dry cough, headache, and pneumonia, which are similar to the symptoms caused by human severe acute respiratory syndrome coronavirus (SARS-CoV) [5,6]. To prevent progress of respiratory distress, renal and hepatic failure, or even death, early intervention is crucial for confirmed cases [2,7,8]. So far, the death toll of the COVID-19 pandemic has surged to over 2000 in China. However, there are specific anti-virus drug therapies, and the high-infectivity/ongoing mortality rate of SARS-CoV-2 emphasizes the demand for drug discovery.

Full-genome sequencing and phylogenetic analysis suggested that SARS-CoV-2 is a distinct clade from the beta coronaviruses and shares a high sequence identity with SARS-CoV [3]. The entry of SARS-CoV into cells is mediated by the interaction between spike glycoprotein (S-protein) and the cellular receptor, which has been identified as angiotensin-converting enzyme 2 (ACE2) [9], homological to the angiotensin-converting enzyme (ACE) [10]. Due to the high homology with SARS-CoV, ACE2 was also considered a gateway for SARS-CoV-2. Moreover, it was reported that the binding affinity of the receptor-binding domain (RBD) of SARS-CoV-2 to ACE2 is 10–20 times stronger than that of SARS-CoV RBD [11]. Most recently, the cryo-electron microscopy (cryo-EM) structure of SARS-CoV-2 and full-length human ACE2-B0AT1 complex were analyzed [11,12]. These findings suggest ACE2 is a potential target for anti-SARS-CoV-2 agent development.

On the other hand, ACE2 is a critical negative regulator of renin-angiotensin system (RAS) [13], which exerts a series of protective effects in multiple organs. Notably, patients with severe SARS-CoV-2 are more likely to have underlying comorbidities, including hypertension, diabetes, cardiovascular disease, and cerebrovascular disease [2]. Inhibition of ACE2 may affect its important protective functions in the lung, kidney, and cardiovascular system. Herein, we reviewed the two-way switch role of ACE2 in the treatment of SARS-CoV-2 pneumonia. This could be informative for targeting the ACE2 therapeutic strategy and the clinical application of RAS drugs for SARS-CoV-2 therapy.

## 2. Function and Distribution of ACE2

**Figure 1:**
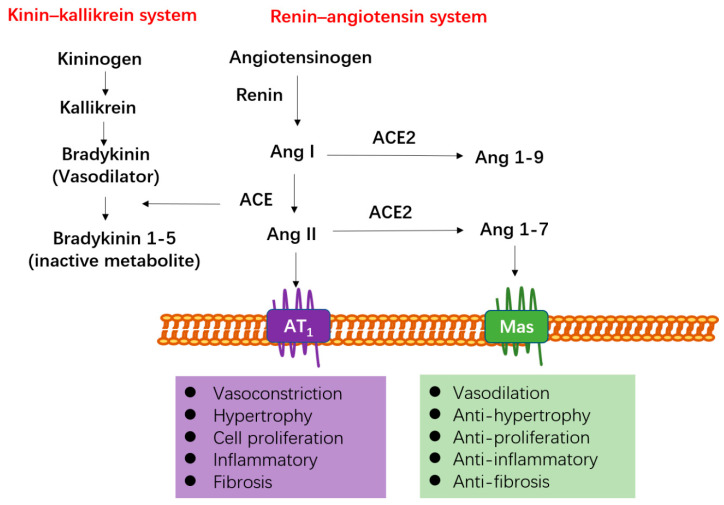
The regulatory mechanism of ACE and ACE2. ACE and ACE2 are both type I membrane-anchored zinc dipeptidyl carboxypeptidase responsible for the cleavage of multiple substrates, including vasoactive peptides. ACE and ACE2 could be considered to act as counterbalances in RAS. ACE participates in the production of the vasoconstrictor Ang II, exerting its effects by binding to G-protein coupled receptor named AT1. ACE2 engages in the conversion of Ang II to Ang 1–7 and Ang I to Ang 1–9, which is much lower efficiency than conversion to Ang 1–7. Ang 1–7 binds to the Mas receptor and a seven transmembrane G-protein-coupled receptor. Ang 1–7 produces vasodilation, anti-hypertensive, anti-proliferation, anti-inflammatory, and anti-fibrotic effects, which is opposed to the actions mediated by Ang II. ACE also exerts important effect on the kinin–kallikrein cascade. ACE converts the strong vasodilator bradykinin, to bradykinin 1–5, which is an inactive metabolite. Thus, ACE is a prominent regulator in blood pressure through RAS and kinin–kallikrein as well.

The ACE2 gene lays on chromosome Xp22 and contains 18 exons, many of which closely resemble exons in the ACE gene. Both ACE and ACE2 are type-I membrane-bound glycoprotein with a single carboxy-terminal extracellular domain, functioning as crucial regulator of the RAS and participating in the cleavage of a diverse set of substrates [14]. As shown in Figure 1, angiotensinogen generated in the liver is transformed into angiotensin I (Ang I) via the action of renin. Then, Ang I is converted to the potent vasoconstrictor angiotensin II (Ang II) by ACE, and Ang II plays important roles in vasoconstriction and aldosterone secretion predominantly through binding to the seven transmembrane G-protein-coupled receptor, named angiotensin receptor type 1 (AT1) [15]. Ang II can also induce vasodilatory effects by binding to angiotensin receptor type 2 (AT2), which has low levels of expression in the cardiovascular system [15]. Therefore, ACE inhibitors (ACEIs) and AT1 receptor blockers (ARBs) are applied for the treatment of cardiovascular diseases via blocking and/or reducing the adverse effects of Ang II [16].

ACE also exerts important effects on the kinin–kallikrein cascade. ACE converts the strong vasodilator bradykinin to bradykinin 1–5, which is an inactive metabolite. Therefore, ACE is a prominent regulator in blood pressure through RAS and kinin–kallikrein as well [17,18]. ACE2 is homologous to ACE and plays a role in the transformation of Ang II to angiotensin-(1–7) (Ang 1–7), and also transforms Ang I to angiotensin-(1–9) (Ang 1–9) [17,18]—meaning much lower efficiency than transformation to Ang 1–7 (Figure 1). Ang 1–7 is mainly generated from the conversion of Ang II by ACE2. Ang 1–7 binds to the Mas receptor, triggering intracellular signal transduction pathways [19]. Ang 1–7 exerts anti-hypertensive, anti-thrombotic, anti-fibrotic, and anti-inflammatory effects, which is opposed to the regulatory mechanism of Ang II [20,21,22,23] (Figure 1). Therefore, ACE and ACE2 could be considered to act as counterbalances in RAS. 

**Figure 2:**
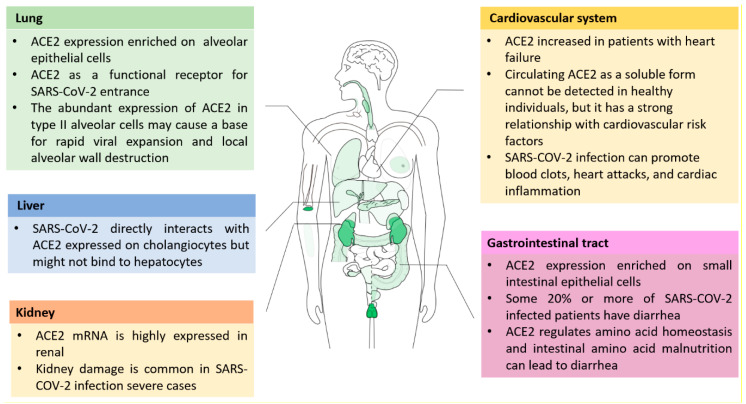
ACE2 tissue distribution and its potential association with severe acute respiratory syndrome coronavirus 2 (SARS-CoV-2) infection sites and disease pathology. ACE2 is mainly localized on endothelial cells and can be found in tissues, and among them kidneys, testis, and gastrointestinal tract, and have the highest ACE2 expressions. SARS-CoV-2 mainly spread via the respiratory tract, and type I and type II alveolar cells are the important sites of entrance for SARS-CoV-2, in particular, the abundant expression of ACE2 in type II pneumocytes may cause rapid viral expansion and local alveolar wall damage, resulting in further severe diffuse alveolar destruction. Except for the lungs, SARS-CoV-2 infection also can extend to many organs, including the heart and blood vessels, kidney, liver, and gut.

ACE2 exerts a series of protective effects in heart function, hypertension, diabetes, kidney, lung, and liver injury [13,18,24,25]. ACE2, as a membrane-bound ectoenzyme, is mainly localized on endothelial cells, and can be identified in tissues, including the kidney, liver, lungs, stomach, intestine, pancreas, testes, and so on [25,26]. SARS pathogen can trigger acute severe lung failure and acute respiratory distress syndrome (ARDS). ACE2, as a functional receptor for SARS-CoV-2, plays a critical role in SARS-induced lung injury [27,28]. ACE2 tissue distribution indicates some association with SARS-CoV-2 infection sites and disease pathology (Figure 2). Although compared with kidney and intestinal epithelia, ACE2 is moderately expressed in the lung, but respiratory symptoms are most severe [29]. SARS-CoV-2 mainly spreads via the respiratory tract, and type I and type II alveolar cells are the important sites of entrance for SARS-CoV-2, in particular, the abundant expression of ACE2 in type II pneumocytes may cause rapid viral expansion and local alveolar wall damage, resulting in further severe diffuse alveolar destruction [30]. Except for lungs, a SARS-CoV-2 infection also can extend to many organs, including the heart and blood vessels, kidney, liver, and the gut [27,30]. SARS-CoV-2 attacking the heart and blood vessels was widely reported that heart damage in nearly 20% of patients out of 416 hospitalized for SARS-CoV-2 in Wuhan, China, and 38% SARS-CoV-2 infected patients out of 184 in a Dutch intensive care unit (ICU) had blood clots, which can break apart and land in the lungs, blocking vital arteries. ACE2 expression was mainly expressed on the surface of small intestinal epithelial cells [31]. More evidence suggested that SARS-CoV-2 can infect the lower gastrointestinal tract, where ACE2 expression is rich, and some 20% or more of patients have diarrhea [27,30]. ACE2 plays an important role in the regulation of dietary amino acid homeostasis, expression level of antimicrobial peptides, and gut microbial ecology [31]. Hashimoto T, et al. suggested that intestinal amino acid malnutrition via ACE2 can cause intestinal inflammation and diarrhea [31]. Different degrees of liver damage were implicated in SARS-CoV-2 infected patients, but its related mechanisms have not been clear. A recent study showed SARS-CoV-2 directly interacts with ACE2 expressed on cholangiocytes, but might not bind to hepatocytes [8]. Recently, it was suggested renal impairment is a common characteristic in SARS-CoV-2 infected patients, which may lead to multiple organ failure and lethal disease [32]. Hence, liver and renal function monitoring, and special care, should be performed in treating SARS-CoV-2 patients.

ACE2 is a functional receptor on cell surfaces for SARS-CoV-2 infection, it also sheds into the plasma as soluble form. The significant increase of circulating ACE2 is detected in the plasma of patients with cardiovascular disease, hypertension, diabetes, and chronic kidney disease [33,34,35,36]. The metalloproteinase ADAM metallopeptidase domain 17 (ADAM-17) is responsible for mediating ACE2 shedding from the cell membrane-bound domain, which can be promoted by Ang II, and release the ACE2 soluble form in plasma [37]. Recently, it was reported that plasma soluble ACE2 in two large and independent cohorts with heart failure were higher in men than in women, and subjects with higher risk for severe SARS-CoV-2 had higher soluble ACE2, which suggested soluble ACE2 may be a marker of severity of SARS-CoV-2 [38].

More evidence, across countries, suggest the mortality rate from SARS-CoV-2 in men is higher than women, which indicates the presence of a potential sex-dependent susceptibility. ACE2 may participate in this different predisposition. The ACE2 gene is located on the X-chromosome, so it gives females a potential heterozygosity compared to males, who are definitely hemizygous [14]. In females, X-heterozygous alleles can activate a mosaic advantage, as well as a greater sexual dimorphism, which may favor them when it comes to suppressing the SARS-CoV-2 infection and local inflammation leading to severe outcomes. In addition, SARS-CoV-2 receptor ACE2 and co-receptor transmembrane serine protease 2 (TMPRSS2) are co-expressed in prostate epithelial cells [39]. TMPRSS2 is highly expressed in human prostate epithelial cells and its regulation by androgen, which may lead to prostate susceptible to SARS-CoV-2 infection, and may be a biological risk factor for males. In addition, it was also reported that the androgen receptor (AR) can bind to the gene transcription promoter sequences of ACE2 and TMPRSS2, and androgen-AR activation may induce the expression of ACE2 and TMPRSS2 [40]. Dihydrotestosterone (DHT, AR activator) induces the expression of ACE2 and TMPRSS2 in activin-sensitive prostate cancer cells (LNCaP) and A549 non-small cell lung cancer cells, and GT0918 (a new generation of AR antagonists) can inhibit DHT-induced expression of ACE2 and TMPRSS2 [40]. These results indicate that AR plays a role in controlling the expression of ACE2 and TMPRSS2. However, this is primarily in experimental models, and there is still no conclusive evidence in humans. 

## 3. Inhibiting ACE2 to Block the Entry of Coronavirus

### 3.1. ACE2 as the Gateway of SARS-CoV-2

**Figure 3:**
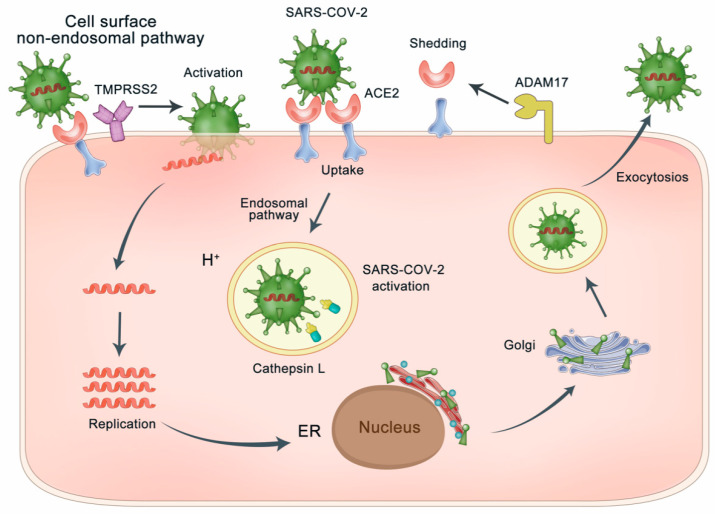
SARS-CoV-2 enters the host cell using the endosomal pathway and/or the cell surface non-endosomal pathway. The endosomal pathway is through ACE2–spike protein interaction, which triggers endocytosis of virus particles through internalization with the ACE2, further mediating the fusion of virus and host cells. In addition, the cell surface non-endosomal pathway for SARS-CoV-2 spike protein priming by TMPRSS2 for viral entry into the cell. ADAM-17 mediated shedding of ACE2 at the cell surface is an important pathological outcome of SARS-CoV-2 infection. TMPRSS2 competes with the metalloprotease ADAM-17 for ACE2 ectodomain shedding. Cleavage of ACE2 by TMPRSS2 enhances the S-protein-driven viral entry.

ACE2 was considered as the functional and essential receptor for SARS-CoV infections. S-protein on the SARS-CoV regulates ACE2 receptor recognition and cellular membrane fusion [41]. In the phase of viral maturation, S-protein is cleaved into two separate subunits, S1 and S2, but for mature SARS-CoV virions, S-protein is not cleaved [42,43], whereas it still has two functional regions, S1 region and S2 region [9]. The RBD at the S1 region directly binds to ACE2 [3], and the S2 region participates in membrane fusion by priming by cellular protease. RBD of S1 region defined at residues 318–510 binding to ACE2 has a stronger affinity than the whole S1 region [9,42,43,44]. The alignment of the RBD from SARS-CoV-2 and SARS-CoV shows a 73.5% sequence identity [11,12]. It was reported that the S-protein of SARS-CoV-2, the same as SARS-CoV, binds to ACE2 and extends into the pocket of the ACE2 catalytic domain [3,12]. As shown in Figure 3, the interaction of ACE2 and S-protein triggers virus particle endocytosis, further mediating the fusion of virus and host cytomembrane. S-protein priming not only employs endosomal cysteine proteases, but also utilizes the cellular serine protease TMPRSS2 on cell membrane. S-protein priming by host cell proteases play an essential role in viral membrane fusion [43,44,45]. The most recent studies suggested that TMPRSS2 was necessary for S-protein priming of SARS-CoV-2, and camostat mesylate, a serine protease inhibitor, could block the activity of TMPRSS2 [46,47,48]. ADAM-17 mediated shedding of ACE2 at the cell surface is an important pathological outcome of SARS-CoV-2 infection [49]. ADAM-17 activity is upregulated as the binding of SARS-CoV to ACE2 and contributes to viral entry [49]. TMPRSS2 was found to compete with the metalloprotease ADAM-17 for ACE2 ectodomain shedding. Cleavage of the C-terminal segment (especially residues 697–716) of ACE2 by TMPRSS2 enhances the S-protein-driven viral entry [50].

Although morbidity between men and women is approximately the same, more evidence across countries indicated the different susceptibility in men and women population. Recently, it was reported that an elevated androgen level increases the risk of SARS-CoV-2 susceptibility and severity in males. Androgen signaling inhibition reduces infectivity of SARS-CoV-2 in human embryonic stem cell (hESC)-derived lung organoids via decreasing the expression of ACE2 and TMPRSS2 levels [51].

### 3.2. Blocking the Binding of S-Protein Binding Site of ACE2 with SARS-CoV-2

The crystal structure of the ACE2 extracellular domains refers to peptidase domain (PD) and collectrin domain. In both SARS-CoV-2 and SARS-CoV, RBD of their S1 region is responsible for interaction with the PD of ACE2, and their interactive affinity was associated with the efficiency of virus infection [11,12]. The variation of RBD of S1 region changes may be important for the adaptive capacity of SARS-CoV-2 and SARS-CoV to infect human cells. In particular, during the 2002–2003 SARS-CoV outbreak, alteration of residues 479 and 487 at RBD proved to be critical to the interactive affinity with human ACE2 via binding to two virus-binding hotspots 31 and 353 on ACE2, center on Lys 31 and Lys353, respectively [52,53]. The hotspots are critical for coronavirus binding as they involve two lysine residues that need to be accommodated properly in hydrophobic environments. Lys31 and Lys353 can form a salt bridge with Glu35 and Asp38, respectively, hidden in the hydrophobic environment [53,54].

Most recently, the receptor recognition by SARS-CoV-2 was also analyzed [12,50,55]. The overall structure of the SARS-CoV-2/ACE2 complex is very similar to the previously reported structure of SARS-CoV interaction with ACE2 [55]. Compared with the SARS-CoV RBD, the two virus-binding hotspots (hot spot-31 and hotspot-353) at the RBD/ACE2 interface are more stabilized due to several residue changes in SARS-CoV-2 RBD [50]. In particular, Gln493 and Leu455 of SARS-CoV-2 RBD stabilizes hotspot-31, and Asp501 of SARS-CoV-2 RBD stabilizes hotspot-353, respectively [50]. In addition, compared with SARS-CoV, four-residue motif (residues 482–485: Gly-Val-Glu-Gly) of SARS-CoV-2 RBD contains structural changes in the ACE2-binding ridge to become more compact interaction with the N-terminal helix of ACE2 [50]. Moreover, the replacement of Val404 to Lys317 in SARS-CoV-2 RBD may lead to tighter association because of the salt bridge formation between Lys317 and Asp30 of ACE2 [12]. The change from Leu472 to Phe486 appears to make stronger van der Waals contact with Met82 [12]. 

ACE2 activity can be inhibited by metallopeptidase inhibitor, MLN-4760, in the low nanomolar range, and lose its enzymatic activity in the concentration of 100 nM inhibitor, but 100 nM MLN-4760 did not have influence on S-protein-induced infection [56]. Towler et al. also reported that the distance alteration among α helix 1 and β-sheet 5 of key residues on S-protein-binding site is less than 0.4A° with or without inhibitor bounded [56]. Therefore, the interaction of S-protein with ACE2 is not disturbed by ACE2 inhibitors or substrates induced conformational alteration. Moreover, the enzymatic activity of ACE2 does not be interfered with by S-protein binding [52].

Recently, the glycosylation sites were considered important for receptor recognition and virus entry [12,52]. In the vicinity of the RBD, three glycosylation sites on PD were observed, including Asn90, Asn322, and Asn53 [12]. It was reported that chloroquine can inhibit SARS-CoV infection via interfering with ACE2 terminal glycosylation [57]. It was still not clear about the specific engagement of sugar moieties in S-protein binding. Taken together, targeting ACE2 at S-protein-binding site, especially hot spot-31 and hot spot-353, is a potential strategy for inhibiting the entry of SARS-CoV-2. Based on molecular docking and structure–activity study, Huentelman MJ, et al. identified a novel ACE2 inhibitor, NAAE (*N*-(2-aminoethyl)-1 aziridine-ethanamine), could modulate ACE2 catalytic activity, and inhibit SARS-CoV S-protein-mediated cell fusion in vitro [58]. On account of the critical segments of ACE2, analogous peptide comprised of two discontinuous segments of ACE2 (a.a. 22–44 and 351–357) artificially linked together by glycine, were synthesized and evaluated, which also exhibited potent anti-SARS-CoV activity in vitro with the half maximal inhibitory concentration (IC_50_) of about 0.1 mM [9]. Recently, it was reported that SARS-CoV-specific human monoclonal antibody, CR3022, could bind potently with SARS-CoV-2 RBD with the calculated affinity (K_D_) of 6.3 nM [59]. Considering the difference in the RBD of SARS-CoV and SARS-CoV-2, it is still necessary to develop novel small molecules or monoclonal antibodies that could bind specifically to SARS-CoV-2 RBD.

## 4. Activating the Peptidase Function of ACE2 for Lung Protection

### 4.1. ACE2 Functions as a Negative Regulator of RAS

Due to the fact that ACE2 counterbalances the effect of Ang II, ACE2 has been considered a negative regulator in RAS. Cardiac contractility defects were observed in ACE2 knockout mice, accompanied by the increase of Ang II [18]. The deficiency of ACE2 also resulted in the inflammatory response in the myocardial infarction regions, triggering neutrophilic infiltration, increasing of interferon γ (IFNγ), and interleukin-6 (IL-6), and activating the downstream extracellular signal-regulated kinase 1/2 (ERK1/2) and c-Jun N-terminal kinase 1/2 (JNK1/2) pathways [60]. ACE2 also exerts protective effects in renal disease through inhibiting oxidative stress, renal inflammation, and fibrosis [61].

Emerging evidence showed the protective role of ACE2 in lung pathophysiology was associated with attenuating RAS. ACE-2 cleaves Ang II to generate Ang 1–7 and thereby limits Ang II accumulation. By reducing the level of Ang II, ACE2 exerts protective effect on lung injury and lung fibrosis [13]. In lung biopsy samples from idiopathic pulmonary fibrosis (IPF) patients, ACE2 mRNA level and enzyme activity were severely decreased, which showed similarly in the lungs of bleomycin-treated rats and C57-BL6 mice [62]. With intratracheal administration of a competitive inhibitor of ACE2, peptide DX600, or ACE2-specific siRNAs, the profibrotic peptide Ang II level increased and the experimental lung fibrosis was enhanced [62]. Purified recombinant human ACE2 systemically attenuated bleomycin-induced lung fibrosis and restrained the progression of lung injury in ACE2 knockout mice [62,63]. Moreover, the upregulation of ACE2 attenuates pulmonary arterial hypertension (PAH) [64], bleomycin-induced pulmonary fibrosis [63]. Except for chronic lung disease, ACE2 is also a protective regulator in acute lung injury (ALI) and respiratory distress syndrome. In the lipopolysaccharide (LPS)-induced ALI models, the expression of ACE2 was reduced, which caused inflammatory injury and lung pathological injury via abnormal activation of toll-like receptor 4 (TLR4) pathway [64]. After intratracheal administration of recombinant ACE2, lung function and inflammatory injury were significantly recovered, accompanied by inhibition of TLR4 pathway [65]. ACE2 not only plays an important role in a SARS coronavirus-induced lung injury, but also in lethal avian influenza A (H5N1, H7N9) induced ALI [66,67]. Yang P.H., et al. reported that H7N9 virus-induced ALI leads to the remarkable downregulation of ACE2, further aggravating lung pathogenesis markedly [67]. These investigations proved the protective roles of ACE2 in lung pathophysiology. Therefore, recombinant soluble ACE2 could be a potential therapy option for acute lung injury, which is now being tested in clinical trials [68,69].

### 4.2. Stimulating the Expression of ACE2

Transcriptional regulation of ACE2 could be effected dynamically under different physiological and pathological conditions (Table 1). Although transcriptional regulation of ACE2 is still unclear, accumulating evidences suggested that Ang II was able to up-regulate ACE and downregulate ACE2 [70]. It was reported that the AT1 receptor-mediated ERK/p38 mitogen-activated protein (MAP) kinase signaling pathway contributed to the downregulation ACE2 expression-induced Ang II, indicating an ACE/ACE2 imbalance in cardiovascular and renal injury [70]. The nuclear factor-κB (NF-κB) signaling pathway is also a key mechanism by which Ang II downregulates ACE2 expression [71]. The relationship between ARBs and ACE2 has been widely investigated, indicating that olmesartan, losartan, telmisartan, and azilsartan could increase the ACE2 mRNA or protein expression levels in animal models of heart diseases [72]. In patients with chronic kidney disease, it was suggested that olmesartan showed unique effects on the upregulation of urinary ACE2 level, which could preserve or improve of renal functions [73]. There are poor reports that ACEIs affect the expression of ACE2 in the heart and the kidney [74,75,76,77]. In normal rats, lisinopril has been shown to modestly increase cardiac ACE2 mRNA levels, while in the myocardial infarction model, valsartan, ramipril, and their combination have no effects on cardiac ACE2 expression [32]. However, enalapril could attenuate downregulation of ACE2 in myocardial infarcted rats [74] and prevent cardiac hypertrophy and dysfunction. It should be noted that no clinical data exist regarding the effects of ARBs and ACEIs on human tissue ACE2 expression or activity in vivo [75,76,77]. 

Except for RAS, the mineralocorticoid receptor blockade (e.g., spironolactone) exerted its beneficial effects by suppressing oxidative stress and increasing ACE2 mRNA in macrophage by increasing the generation of Ang 1–7 and decreasing the formation of Ang II [78]. Recent evidence has suggested that vitamin D, a fat-soluble-vitamin, plays an essential role in the immune response against viral infections, and indicated vitamin D is a negative endocrine RAS modulator [79,80,81]. It can induce ACE2/Ang-(1–7)/MasR axis activity and inhibit renin and the ACE/Ang II/AT1R pathway, thereby upregulating expression level of ACE2, MasR, and Ang-(1–7), and exerting a potential protective role against ALI and ARDS [81]. Vitamin D deficiency also increases susceptibility to viral infections as well as the risk of recurrent infections [80]. Therefore, vitamin D may be a potential therapeutic approach to combat SARS-CoV-2 infection induced ARDS [80,81].

In addition to Ang II, apelin is another catalytic substrate for ACE2 and is considered an inotropic and cardioprotective peptide [82,83]. Among apelin peptides, apelin-13 is the most abundant in human plasma and cardiac tissue, and it exists predominantly under its pyroglutamylated form (Pyr-apelin-13) [84]. ACE2 could cleave and inactivate Pyr-apelin-13. In ACE2 knockout mice, hypotensive action of Pyr-apelin-13 was enhanced, with higher apelin levels in plasma. In turn, apelin-13, via activation of G protein–coupled receptor, the apelin receptor (APJ), increased ACE2 promoter activity in vitro, and upregulated ACE2 expression in failing hearts in vivo [83]. Moreover, apelin-13 treatment also increased cardiac contractility and ACE2 levels in AT1R-deficient mice, which demonstrate an antagonistic relationship between the RAS and apelin [83].

There also exists other molecules in the transcriptional regulation of ACE2 level. Sirtuin 1 (SIRT1) could regulate ACE2 level via AMP-activated protein kinase (AMPK) signals and this role might be beneficial to their protective role against cellular stress in type 2 diabetes mellitus (T2DM), diabetic nephropathy, and myocardial injury [85,86]. Resveratrol has multiple beneficial activities, including antioxidant, anti-inflammatory, and cardioprotective effects [87]. It was reported that resveratrol administration could increase SIRT1 and ACE2 protein expression to reduce oxidative stress and modulate of RAS [88]. The cytokines showed different functions in regulating the expression of ACE2. IL-1β upregulated ACE2 transcription, in ARDS, IL-1β exerted protective effects and promoted epithelial repair, which was likely referred to the regulation of ACE2 in several inflammatory conditions [85]. Whereas, IL-4 downregulated expression of the SARS-CoV receptor ACE2 in Vero E6 cells [89]. The downregulation of ACE2 expression through addition of IL-4 might suggest a novel antiviral strategy against SARS-CoV infection. ACE2 was recently reported to be a human interferon stimulated gene (ISG) in airway epithelial cells [90]. Interferon a (IFNα), and to some extent IFNγ, led to the upregulation of ACE2 expression in the main human upper airway cells [90]. As SARS-CoV-S caused ACE2-receptor-mediated internalization, the host IFN response could enhance the capability for SARS-CoV and SARS-CoV-2 to maintain cellular targets in the near upper airway epithelial cells [90], which indicated the clinic usage of IFNα might aggravate the SARS-CoV-2 infection.

### 4.3. The Clinical Usage of ACEIs and ARBs for SARS-CoV-2 Infected Patient with Cardiovascular Diseases: Pros and Cons

In a descriptive study of the 99 patients with SARS-CoV-2 pneumonia, 50 (51%) patients had chronic diseases, and 40 (40%) patients had cardiovascular and cerebrovascular diseases [91]. Given that ACE2 is a gateway for SARS-CoV-2, binding of the SARS-CoV-2 virus to its ACE2 appears to lead to an imbalance in RAS signaling: a decrease in beneficial products derived from ACE2 relative to enhance the potentially harmful effects from Ang II/AT1R1 action. In particular, the dysregulation of RAS is recognized as a main factor in the progression of cardiovascular pathologies [92,93], so the problems of ACEIs or ARBs administration for SARS-CoV-2 infected patients with cardiovascular diseases are subject to address in clinical practice.

Because ACE and ACE2 exhibits structural difference in their active site, classical ACE inhibitor cannot inhibit the activity of ACE2 [94]. On the one hand, it was suggested that ACE or AT1R inhibitors could still be used for SARS-CoV-2 infected patients with cardiovascular diseases. Firstly, pathological findings of SARS-CoV-2 were related to ARDS [95], and the clinical symptoms of SARS-CoV-2 were highly similar to those of SARS coronavirus infection [96,97]. The upregulation of Ang II mediated by ACE was observed in ARDS pathogenesis, which caused severe lung failure via the interaction of Ang II with AT1 receptor [63]. Therefore, AT1R or ACE blockade alleviated pneumonic injury. Secondly, in the lung failure caused by SARS-CoV, the level of ACE2 protein is decreased, but ACE levels are normal [28]. Due to ACE2 as a key negative regulatory factor for acute lung injury, SARS-CoV-induced ACE2 downregulation increase Ang II level, which leads to the severity of lung pathologies [28]. In Spike-Fc–treated mice, ACE2 was pulled down, and its expression was downregulated, while AT1R inhibitors could rescue acute severe lung injury [28]. Hence, SARS-CoV-induced lung injury can be attenuated by AT1R inhibitor. Flow cytometric analysis of peripheral blood of SARS-CoV-2 infected patients implied T cell overactivation with the upregulation of Th17 [95]. The selective antagonist of the AT1 receptor, losartan, prevents the development of ARDS triggered by lung bacterial infection through suppressing the activation of neutrophils by N-formylmethionyl-leucyl-phenylalanine (fMLP) [98].

On the other hand, some clinicians suggested that although ACE2 is insensitive to ACE inhibitors, ACE inhibition or AT1 receptor blockade increased ACE2 gene expression or activity in cardiac and vascular tissue [99,100]. Actually, in the context of renal cortex, ACE2 expression remained unchanged with ACE inhibition or AT1 receptor blockade, but its activity increased with the administration of lisinopril or losartan alone [101]. The same therapeutic agents with the same dose administrated to the same rate for the same period caused varied consequences on ACE2 expression and activity in cardiac and renal tissue [101]. Taken together, these findings suggested a tissue-specific regulation of ACE2 expression under ACE inhibition or AT1 receptor blockade that affects the generation of Ang II [26,101]. Therefore, the ACE or AT1R blockers did not necessarily upregulate ACE2 expression in the lungs, and there was not enough reasons to stop taking ACE or AT1R inhibitors for SARS-CoV-2 infected patients with cardiovascular diseases.

ACEIs and ARBs are differently related to ACE2: ACEIs inhibit ACE in the transformation of Ang I to Ang II and, consequently, reduce pro-inflammatory Ang II production, and potentially decrease the generation of Ang 1–7. However, ARBs through blocking the Ang II interacted with AT1R could divert a larger proportion of generated Ang 1–7 towards ACE2, and increase the generation of Ang 1–7 level, which produces vasodilation, anti-hypertensive, anti-proliferation, anti-inflammatory, and anti-fibrotic effects. Therefore, multiple clinical trials (NCT04335786, NCT04355936, NCT04332666, NCT04312009, NCT04311177, NCT04328012, and NCT04340557) have recently proposed the tentative use of ARBs, such as telmisartan, valsartan, and losartan, as alternative options for treating SARS-CoV-2 patients (Table 2). In these clinical trials, there were some inconformity in the study design, including inclusion and exclusion criteria, dose selection, outcomes management, and biomarkers selection. Virus spike protein binds to ACE2 to form a complex suitable for cellular internalization, which leads to a partial decrease or total loss of the enzymatic function of ACE2 in the alveolar cells, and in turn upregulates the tissue concentration of pro-inflammatory Ang II by reducing its degradation and reducing the concentration of its physiological antagonist Ang 1–7. High levels of Ang II on the lung interstitium can promote apoptosis, initiating a self-powered cascade with release of proinflammatory cytokines, leading eventually to ARDS. Therefore, available AT1R blockers have the potential to block this pathological process mediated by Ang II and the excessive angiotensin-mediated AT1R activation. AT1R blockers may suppresses the development of ARDS and reduce morbidity (admission to ICU) and mortality. Besides ACE or AT1R blockers, there also existed other therapies related to the RAS pathway. Inhaled delivery of ACEIs/ARBs is a potential alternative for the treatment of RAS-mediated acute lung injury in SARS-CoV-2 infected patients [102]. Administration of ANG 1–7 (or TXA127, a purified formulation of ANG 1–7) activating the G protein-coupled receptor Mas (MAS1) or AVE 0991 (nonpeptide MAS1 agonist) could also be considered for SARS-CoV-2 infection with pulmonary damage [102].

Except for focusing on new antiviral agents for SARS-CoV-2 infection, therapeutics that target clinical manifestations, referring to tissue damage, should also be emphasized. ACEIs and ARBs are potential treatments for preventing those complications, and the benefit and risks of these drugs should be assessed in randomized controlled trials. The preclinical studies should also be carried out to elucidate the roles of the RAS pathway in SARS-CoV-2 pathobiology and develop potential RAS-targeted therapeutic strategies.

## 5. Control of the Two-Way Switch of ACE2 in SARS-CoV-2 Infection and Its Underlying Comorbidities

Compared with SARS-CoV, SARS-CoV-2 had comparable transmissibility, which may depend on high affinity to a virus-binding host cell receptor, ACE2, and interaction with host cell protease-cleaving virus S-protein. Because ACE2 is a functional receptor in SARS-CoV-2 infection, targeting ACE2 may be a potential antiviral treatment option for SARS-CoV-2 infection. Ideal ACE2 inhibitor or peptides should act on the S-protein-binding site, and not reduce the peptidase activity of ACE2 for processing Ang II to Ang-(1–7). Therefore, the potential effect of ACE2 inhibitor on RAS should be evaluated, and the level of plasma Ang II level should be determined to prevent the adverse drug reaction related to lung injury, and cardiovascular and kidney disease. Given that SARS-CoV-2 spread depends on ACE2 for entry and TMPRSS2 for S-protein priming, and the co-expression of ACE2 and TMPRSS2 on the same virus target cells [103], like lung AT2 cells, dual blockage of TMPRSS2 and ACE2 could be promising treatment for SARS-CoV-2 infection.

SARS-CoV-2 infection can trigger a cytokine storm and induce autoimmune-related damages. The increase of ACE2, further mediating the upregulation of Ang 1–7 levels and decrease of Ang II, suggested being protective against lung injury. ACE2 has been shown to modulate innate immunity and decrease the level of pro-inflammatory cytokines [13]. Recombinant human ACE2 protein, therefore, can protect individuals with SARS-CoV-2 from developing acute severe lung failure and acute respiratory distress syndrome. Recently, it was reported that SARS-CoV-2 infection could be significantly inhibited by human ACE2 protein in human blood vessel organoids and kidney organoids models, and human ACE2 can neutralize virus pseudotyped with SARS-CoV-2 spike proteins in vitro, which indicates that human ACE2 protein might block the earlier entry of SARS-CoV-2 [104]. Considering that the lung is the major target, lung organoids should also be taken to assess the further protective effects. Further clinical studies are needed to reveal the effect of human ACE2 protein in SARS-CoV-2 infections. The clinical trials of recombinant human ACE2 refers to APN01 (NCT00886353) and GSK2586881 (NCT01597635), which completed the Phase I and Phase II study, respectively. In Phase II, for investigating the intravenous administration of GSK2586881 in patients with acute respiratory distress syndrome, the safety and efficacy were confirmed for its (well) toleration with a wide range of doses, and ACE2-mediated rapid transformation of Ang II to Ang 1–7, as well as tendency to reduce IL-6 levels [105,106]. Because recombinant ACE2 may compete with AT1R for Ang II, the balance of RAS is likely to be influenced by an excess of recombinant ACE2, which is important for the clinical success of recombinant human ACE2. Recombinant human ACE2 is a glycosylated protein, and thus its preparation requires a time- and cost-consuming protein expression system with mammalian or insect cells. A randomized, open label, controlled clinical study of recombinant bacterial ACE2 receptors, B38-CAP, were registered in ClinicalTrials.gov (NCT04351581) to evaluate the therapeutic effect of SARS-CoV-2 infection and lung injury. B38-CAP is a bacteria-derived ACE2-like enzyme that downregulates Ang II levels in mice and suppresses Ang II-induced hypertension, pathological cardiac hypertrophy, and myocardial fibrosis [107]. Treatment with an ACE2-like enzyme in bacteria B38-CAP might be do the same mechanism of rhACE2 in inhibiting SARS-CoV-2 infection [107]. Recently, it was indicated that ACE2 mutations in the N-glycosylation motif (N90 and T92) and buried hydrophobic sites that might have better effects on plasma membrane trafficking could increase S binding, which provides a novel way to increase the affinity to SARS-CoV-2 [108].

In conclusion, ACE2 is a functional receptor in SARS-CoV-2 infection, and it has a high affinity with RBD of the SARS-CoV-2 S-protein. Blocking the S-protein-binding site, especially on hot spot-31 and hot spot-353, with ACE2-derived peptides or small molecule inhibitors, could be a potential treatment option. In addition, a recombinant ACE2 protein could be another treatment option for SARS-CoV-2 infection related severe lung failure and acute respiratory distress syndrome [109]. In SARS-CoV infection, ACE2 was downregulated, further activating the RAS and increasing Ang II, resulting in pathogenesis of acute lung injury or severe ARDS. The molecular relationship between SARS-CoV-2 infection and the expression of ACE2, the role of the RAS in lung failure should be further investigated. In addition, the safety and efficacy of ACE2 inhibitors or recombinant ACE2 protein in patients with SARS-CoV-2 infection also needs to be further evaluated in the preclinical and clinical study.

## Figures and Tables

**Table 1:** The regulation of angiotensin-converting enzyme 2 (ACE2) by different mediators.

Mediator	Effect on ACE2 Expression	Pathway	Associated Disease
AT1 receptor blockers, such as olmesartan, losartan, telmisartan, azilsartan	Upregulation	Renin–angiotensin system; extracellular-signal-regulated kinase/mitogen-activated protein kinase (ERK/MAPK) signaling pathway; nuclear factor-κB (NF-κB) signaling pathway	Acute lung injury, hypertensive cardiovascular and renal damage
ACE inhibitors, such as Lisinopril, Enalapril	Downregulation	Renin–angiotensin system	Myocardial infarction
Vitamin D	Upregulation	Renin–angiotensin system	Acute lung injury (ALI) Acute respiratory distress syndrome (ARDS)
Spironolactone	Upregulation	NADPH oxidase related pathway	Heart failure
Resveratrol	Upregulation	alpha-amino-3-hydroxy-5-methyl-4-isoxazole propionic acid (AMPA) signaling pathway	Diabetes mellitus, cardiac fibrosis and heart disease
Apelin-13	Upregulation	Apelin-the apelin receptor (APJ) activation pathway	Cardiovascular diseases
Interleukin-1β (IL-1β)	Upregulation	Cytokine signaling pathway	SARS coronavirus diabetes mellitus
Interferon α (IFNα), Interferon γ (IFNγ)	Downregulation		SARS coronavirus
Interleukin-4 (IL-4)	Downregulation		SARS coronavirus

**Table 2:** Clinical trials evaluating the efficacy of ACE2-ralated therapy and RAS drugs in SARS-CoV-2 pneumonia treatment.

Clinical Trials Identifier	Study Title	Interventional Drug	Interventional Study Design
NCT04351581	Combination of Recombinant Bacterial ACE2 Receptors -Like Enzyme of B38-CAP and Isotretinoin Could be Promising COVID-19Infection- and Lung Injury Preventing Drug Better Than Recombinant Human ACE2	rbACE2 plus Aerosolized Isotretinoin	Randomized Parallel Assignment Open Label
NCT04355936	Telmisartan for Treatment of COVID-19 Patients	Telmisartan	Randomized Parallel Assignment Open Label
NCT04335786	Valsartan for Prevention of Acute Respiratory Distress Syndrome in Hospitalized Patients with SARS-CoV-2 (COVID-19) Infection Disease	Valsartan	Randomized Parallel Assignment Quadruple (Participant, Care Provider, Investigator, Outcomes Assessor)
NCT04312009	Losartan for Patients with COVID-19 Requiring Hospitalization	Losartan	Randomized Parallel Assignment Quadruple (Participant, Care Provider, Investigator, Outcomes Assessor)
NCT04311177	Losartan for Patients with COVID-19 Not Requiring Hospitalization	Losartan	Randomized Parallel Assignment Quadruple (Participant, Care Provider, Investigator, Outcomes Assessor)
NCT04328012	COVID MED Trial-Comparison of Therapeutics for Hospitalized Patients Infected with SARS-CoV-2	Losartan combined with lopinavir/ritonavir	Randomized Parallel Assignment Double blind, placebo controlled
NCT04340557	Do Angiotensin Receptor Blockers Mitigate Progression to Acute Respiratory Distress Syndrome with SARS-CoV-2 Infection	Losartan	Randomized Parallel Assignment Open Label
NCT04332666	Angiotensin-(1,7) Treatment in COVID-19: the ATCO Trial (ATCO)	Angiotensin 1–7	Randomized Parallel Assignment Triple (Participant, Investigator, Outcomes Assessor)

## Abbreviations

COVID-19	coronavirus disease 2019
SARS	severe acute respiratory syndrome
SARS-CoV-2	severe acute respiratory syndrome coronavirus-2
PHEIC	public health emergency of international concern
S-protein	spike glycoprotein
ACE2	angiotensin-converting enzyme 2
ACE	angiotensin-converting enzyme
RBD	receptor-binding domain
cryo-EM	cryo-electron microscopy
RAS	renin-angiotensin system
Ang I	angiotensin I
Ang II	angiotensin II
AT1	angiotensin receptor type 1
AT2	angiotensin receptor type 2
ACEIs	ACE inhibitors
ARBs	AT1 receptor blockers
ARDS	acute respiratory distress syndrome
ICU	intensive care unit
ADAM-17	ADAM metallopeptidase domain 17
TMPRSS2	Transmembrane Serine Protease 2
AR	androgen receptor
DHT	dihydrotestosterone
LNCaP	activin-sensitive prostate cancer cells
ER	endoplasmic reticulum
hESC	human embryonic stem cell
PD	peptidase domain
NAAE	N-(2-aminoethyl)-1 aziridine-ethanamine
IC_50_	the half maximal inhibitory concentration
K_D_	the calculated affinity
IFN	interferon
IL	interleukin
ERK	extracellular signal-regulated kinase
JNK	c-Jun N-terminal kinase
IPF	idiopathic pulmonary fibrosis
PAH	pulmonary arterial hypertension
ALI	acute lung injury
LPS	lipopolysaccharide
TLR4	toll-like receptor 4
MAP	mitogen-activated protein
ERK/MAPK	extracellular-signal-regulated kinase/mitogen-activated protein kinase
NF-κB	nuclear factor-κB
AMPA	alpha-amino-3-hydroxy-5-methyl-4-isoxazole propionic acid
APJ	the apelin receptor
SIRT1	Sirtuin 1
AMPK	AMP-activated protein kinase
T2DM	Type 2 diabetes mellitus
ISG	interferon stimulated gene
fMLP	N-formylmethionyl-leucyl-phenylalanine
MAS1	the G protein-coupled receptor Mas
